# Can We Afford to Exclude Patients Throughout Health Technology Assessment?

**DOI:** 10.3389/fmedt.2021.796344

**Published:** 2022-01-25

**Authors:** Janet L. Wale, David Chandler, Deborah Collyar, Dominique Hamerlijnck, Roberto Saldana, Zack Pemberton-Whitely

**Affiliations:** ^1^HTAi Patient and Citizen Involvement Interest Group (PCIG) Chair, Brunswick, VIC, Australia; ^2^Psoriasis and Psoriatic Arthritis Alliance (PAPAA), St Albans, United Kingdom; ^3^Patient Advocates in Research (PAIR), Danville, CA, United States; ^4^Patient Expert European and Dutch Lung Foundation, Amsterdam, Netherlands; ^5^Spanish Platform European Patients' Academy on Therapeutic Innovation (EUPATI), Madrid, Spain; ^6^Acute Leukemia Advocates Network and Leukaemia Patient Advocates Foundation, Bern, Switzerland

**Keywords:** patient involvement, patient engagement, health technology assessment, value, person-centered, patient-reported outcomes, patient preference studies

## Abstract

Health technology assessment (HTA) is intended to determine the value of health technologies and, once a technology is recommended for funding, bridge clinical research and practice. Understanding the values and beliefs expressed by patients and health professionals can help guide this knowledge transfer and work toward managing the expectations of end users. We gathered patient and patient group leader experiences to gain insights into the roles that patients and patient advocacy groups are playing. We argue that through partnerships and co-creation between HTA professionals, researchers and patient advocates we can strengthen the HTA process and better align with service delivery where person-centered care and shared decision making are key elements. Patient experiences and knowledge are important to the democratization of evidence and the legitimacy of HTAs. Patient preference studies are used to balance benefits with potential harms of technologies, and patient-reported outcomes (PROs) can measure what matters to patients over time. A change in culture in HTA bodies is occurring and with further transformative thinking patients can be involved in every step of the HTA process. Patients have a right to be involved in HTAs, with patients' values central to HTA deliberations on a technology and where patients can provide valuable insights to inform HTA decision-making; and in ensuring that HTA methodologies evolve. By evaluating the implementation of HTA recommendations we can determine how HTA benefits patients and their communities. Our shared commitment can positively effect the common good and provide benefits to individual patients and their communities.

## Introduction

The COVID-19 pandemic has brought about many changes in our healthcare systems. Some of these can provide benefits for patients, such as widespread use of telemedicine and decentralized clinical trials (1). We have also seen many shortcomings regarding access to medicines and vaccines; and how we get more evidence and context to decision makers, clinicians and the public. Regulators, health technology assessment (HTA) bodies, payers and industry have learned the value of aligning their processes, engaging with each other and creating more opportunities for international cooperation (2). Better alignment can convey information to decision makers in a timely fashion and assist them in dealing with uncertainty and change (2). The Medicines and Healthcare products Regulatory Agency (MHRA) as the UK regulator for market access has worked with patient advocates to develop and release its first patient involvement strategy (3), placing it in line with, for example, the European Medicines Agency (EMA) (4) and U.S. Food & Drugs Administration Agency (FDA) (5).

HTA is intended to bridge clinical research and clinical practice, and to determine the value of health technologies (6, 7). Understanding the values and beliefs expressed by patients and health professionals can help guide knowledge transfer from clinical trials to practice, and work toward matching the realities and expectations of end-users (8). Over recent years we have seen a progression from “should we involve patients” in HTAs (9), “can we afford to involve patients” (10), and a “call to action” (11) to the present situation with COVID-19 that indicates we cannot afford to leave patients out of HTAs (see Table 1). Partnership approaches are important to keep HTA aligned with the rest of the healthcare system where person-centered care and shared decision-making are key elements [e.g., (12)].

**Table 1:** Key messages calling to strengthen patient involvement in health technology assessment.

**Arguments for why patients should be involved in health technology assessments (9)** From a patients' rights perspective, patients have a right to participate in the planning and delivery of their health care, where HTA determines the health services, procedures and technologies available to them; building trust in the health system. Value to patients is central to HTA deliberations and to healthcare systems. Centering on evidentiary contributions, patients can provide valuable insights to inform HTA decision-making. From a methodological perspective, patients can help HTA methodologies to evolve.
**Call to action for HTA agencies and all stakeholders to work together for meaningful patient involvement (11)** Goal 1: Working together with shared purpose. Goal 2: A change in HTA culture, with integration of patient involvement. Goal 3: Alignment with HTA agency goals, to improve health outcomes – and a positive impact on the diverse populations served. Goal 4: Patient involvement at every step of the HTA process. Goal 5: Transformative thinking that involves patient leaders, with use of a unifying language.
**Can we afford to exclude patients throughout health technology assessments (present paper)** These steps are needed to ensure better use of healthcare spending: 1: Bring HTA in line with other parts of the healthcare system - we need to work as partners and co-create patient involvement in HTA. 2: Increase transparency and trust in technology development, regulation and funding informed by HTA - we all need to be honest about our different biases. 3: Activate an awareness and accountability system for how technologies are used in healthcare systems.

## Involving Patients in HTA

HTA addresses important questions that patients and clinicians share, including: does the technology work? If so, for whom, how well, and at what cost? Does it provide value for individual patients and the health system, does it fit within care pathways, and is it worth funding, largely on the basis of “cost” (7)? A health technology includes drugs, diagnostic tests, medical devices and healthcare procedures. HTA is defined on the basis of scientific rigor and evidence, with multi-stakeholder deliberations to appraise the evidence (13). The patient perspective is important in the democratization of evidence. Co-creation with patients can add to the legitimacy of the HTA process for the common good (14). Currently patient involvement is limited in its scope and barriers to their involvement include the lack of information to patients and public about HTA and the lack of policies (15–17), and the need for culture change (18–21). The “invited spaces” for patient participation have been set by HTA policy and practice and leave significant opportunities for broadening through mutual discussions (22). Public representatives have a place on the appraisal committees in a number of countries (17, 23, 24) and in some healthcare systems patients are payers in addition to being the focal point of what healthcare is about. As payers, patients are legitimate stakeholders within HTA. Their role as a payer can cause financial distress for patients (25). HTA is therefore an important methodology for health systems to make decisions on what services and technologies are funded and for universal health coverage (26).

Public awareness of healthcare has grown as a result of the COVID-19 pandemic, particularly in how infectious diseases spread, public health preventive actions, vaccine development, adverse effects of health technologies, regulatory processes, and the availability and distribution of protective clothing, medical interventions and vaccines. Healthcare systems have been stretched in many ways, including their capacities, access to equipment and technologies aggravated by arguments about how the disease is spread, use of face masks, and the science (27). These health system stresses have often meant that patient-centered healthcare has been side-lined, leaving people in critical conditions without their loved ones around them (28). Communication and support, including access to digital technologies, can be limited particularly for marginalized and vulnerable population groups (1).

In its June 2021 position statement, the International Network of Agencies for Health Technology Assessment (INAHTA) stated that “Patient involvement is recognized by INAHTA as an important and valuable element in the conduct of HTA” (29). In a plenary session of the HTAi 2021 Annual Meeting on “Patients at the Heart of Innovation” a call was made for person-centered HTA (30). There appears to be consensus that “we can do more, and do better.” HTA bodies calling for patient input often rely on patient groups to provide input that helps inform deliberations (24). Once received, patient input can be difficult to incorporate into the committee papers and formal assessment (20, 31, 32). Discussions are taking place to overcome at least some of these barriers (33). On occasion, the information provided fills a gap in knowledge or understanding of the appraisal committee (34, 35). And patients can make a difference as evidenced by an example of an assessment in sickle cell anemia for the Institute for Clinical and Economic Review (ICER). The sickle cell patient community highlighted “the appalling trade-off between choosing to manage intolerable pain from home or choosing to go to the emergency room, where many are met with racial prejudice, uninformed medical professionals, and a constant need to advocate for adequate pain management.” Patients needed to meet a prior authorization requirement for opioid pain management, which hinders their access (https://icer.org/wp-content/uploads/2020/10/ICER_SCD_Response-to-Comments_031220.pdf). This raises the issue of need to optimize overall healthcare when providing a medicine or technology for treatment of a health condition.

## Are We Seeing Changes in Approach?

HTA professionals are looking to more “scientific” ways to provide patients' perspectives, as with syntheses of qualitative studies to provide patient evidence (36). Patient preference studies are now being extended beyond economic studies to build clinical trial evidence for an intervention or technology (37–40). Uptake in the USA has been slow (41, 42). In Europe however the Innovative Medicines Initiative (IMI) funded PREFER (https://www.imi-prefer.eu/about/) project involves patient groups to provide guidance for industry, regulatory authorities and HTA bodies on how and when to include patient preference studies on benefits and risks of medicines. The PREFER framework covers validated patient-reported outcome measures (PROMs), clinician-reported outcomes and observer-reported outcomes within a disease setting. These are used to weight the clinical trial evidence. How preference studies relate to patient input into HTA processes and actively involving patient advocates and patient advocacy groups is less clear. The IMI H2O Health Outcomes Observatory project (https://health-outcomes-observatory.eu/) involves patient groups and is creating a data governance and infrastructure model to collect PROMs and incorporate them into healthcare decision making at an individual and population level. Patients have ultimate control of their health data in this project. Qualitative studies that are used to inform patient preference studies are the type of studies that would make PROMs more meaningful and could help individual patients and patient groups better monitor their health conditions and the effects of treatments (43–45). As examples, Janssens et al. (45) showed that for people with multiple myeloma life extension is not the only thing they want from treatment. They want to retain the ability to carry out their daily activities and to maintain independence and mobility. Permanent and severe side-effects and symptoms are of concern to them. ICER noted that the US Food and Drug Administration (FDA) approvals covering drugs for relapsing remitting multiple sclerosis (MS) were based on reductions in the number of relapses. Patients told ICER that accumulating longer-term functional disabilities were the most important outcome for them (ICER HTAi 2021 presentation—personal communication). In an oral presentation at the same meeting, patient advocates highlighted the importance of upper body function and independent living for people with progressive MS who were in wheelchairs (https://youtu.be/hB_eII-b0P8). PROMs are important in capturing “what matters” to patients (46). Patient groups are already forming partnerships to develop apps to personally collect data to monitor and report on their condition, its evolution and the effects of interventions. An example is “Patient Voice – myGUT” in collaboration with Microsoft (personal communication).

Patient-reported data not only has the capacity to empower patients in managing their own health condition but also contributes to broader knowledge that can inform healthcare more generally, including HTAs (47, 48). Patients have felt that they are peripheral to the HTA process and that their involvement takes place too late in the process to make any real difference. Patient involvement is needed early and through all stages of the HTA process from topic selection, scoping, examining evidence, appraisal committee deliberation to determine value, and in formulating recommendations for funding or subsidy (11, 29). An early experience from one co-author, as a “patient expert” at an HTA appraisal, highlights this:

“*I was led into a room with a very large table. Everyone had their heads down and were very intent on what was in front of them. When prompted, I started talking but I was very quickly interrupted and told they had read my “testimonial statement” in the committee papers. They did not need anything more from me... I went there to provide a voice to the voiceless, but left feeling that I had been gagged…”* Patient advocate

This example and others from the literature (11, 23, 32, 49) highlight that we need to address what patients and patient groups are being asked to do in HTA and why.

## How Can we Facilitate Change?

We advocate that collectively, and at all stages of the HTA process, we can integrate the voices of patients, their advocates and support groups. We propose working together to democratize HTA processes, from governance to making recommendations on specific interventions. We see this as a right (9). Frank, comprehensive and respectful conversations are needed with patient advocates and patient advocacy groups about where and how they can provide fruitful, positive and meaningful contributions, and what impacts and benefits these can achieve.

Currently, patient advocates and patient groups may not have a clear understanding of the earlier stages in the development of the technology and in the HTA process, or know if patients or patient groups were involved. They also need clarity about what treatments are already available, at what cost, with what treatment effectiveness, burden and side-effects, and for which sub-groups of patients. Background and landscape analyses can help patients offer more complete understanding and perspectives on the value of a particular technology as they explore and explain the trade-offs that patients must face. Yet scientific and medical jargon can deter patients from joining conversations. Health literacy principles apply and facilitate learning and mutual understanding (22, 50). Patient and public participation should have a direct effect on policy and decisions with inclusion of people's values, ideas and sentiments such that all participants can “live with the result” (22).

Transparency and trust in technology development, regulation and funding informed by HTA can be increased if we all are transparent about our different perspectives, limitations and biases. We also need open discussion on the conflicts of interest of each person involved. For example, HTA appraisal committees may be uncomfortable hearing about individual patient experiences and unmet needs (49), or they may not see the relevance of patients being present.

“*Patients/patient group representatives are often not really listened to when they speak. The expectation is that they just want the new [better] technology, and they are in league with industry anyway.”* Patient advocate

Yet, “*patient advocates tend to change the environment and tenor of the discussion. This gives people on all sides the space to say things they may not normally feel comfortable saying—when “we let them”.”* Patient advocate

An example from ICER shows where a new treatment for sight loss (blindness) failed to achieve traditional measures of cost-effectiveness. Patients and their families conveyed how extensive the benefits of better sight (even partial) are for the entire family through improvements in school, work, and social functioning. ICER developed an alternative economic model incorporating these benefits that was accepted as a reasonable long-term value (HTAi 2021 presentation, personal communication). The value for patient communities needs to be clear. It is also important to understand at the start what the place of the technology is: is it a “breakthrough” technology, another me too, an older product revitalized? This can have an impact on the amount of time patient advocacy groups spend on preparing patient input.

“*On an HTA appraisal committee I was asked why I was not supporting approval of a cheaper, less effective drug. I was able to state very clearly because if approved it would be used and would make it more difficult for patients to access treatments that were much more likely to be effective, and so prolong their discomfort and suffering.”* Patient advocate

“*We need to challenge patient groups to take more responsibility for better outcomes for their patients by insisting that we get better, not just more, treatments. And that they add value to patients' lives without causing them to go bankrupt.”* Patient advocate

“*We should show how we represent a group of patients, not just our own experience. Part of the responsibility of an HTA patient advocate is to give a spectrum of issues and experiences. This approach helps build our credibility, and necessitates our authority as peers with specific expertise on the perspectives of service users.”* Patient advocate

In recent years patient advocates and their organizations have become better informed, educated and trained to concentrate on their patients' experiences and knowledge so to effectively contribute to regulatory and HTA decision-making [e.g., (37, 51–53)]. They are also involved in clinical trial design (54, 55). Now we need to co-create and democratize the evidence (56). Patient advocates and patient advocacy groups need to have access to comprehensive, informative data on the technology they are being asked to comment on, which often does not happen (22). The justification of not sharing the data on a technology is that manufacturers need to protect confidential and proprietary information, and laws on “advertising prescription technologies” to the public that interrupt adequate flows of information (57). A non-disclosure agreement (NDA) is already used by the HTA body for the other members of the committee and can also be used for patients. Clinical trial reports may be behind journal paywalls or not accessible to the public; similarly comparative data, longer-term and real-world data. Some HTAs have tried to resolve these limitations. The Scottish Medicines Consortium “Summary of Information for Patients” (SIP) is a simple summary of clinical trial data for patient groups to be provided by industry as part of its product submission that is being used to develop similar processes in other countries (58). While promising, the authors of this paper are concerned that industry may essentially control what patient advocacy groups know about the new technologies.

“*We are “selling” something to patients without giving them the background information and evidence-base that they need to be able to make rational choices/judgements.”* Patient Advocacy Group

Germany's Federal Joint Committee (G-BA) ensures that its patient advocates receive full information (59), HTAi 2019 PCIG workshop—personal communication], demonstrating that “political commitment” can overcome these information barriers (22). “Partnership synergy” is the ability to work together by combining resources in order to produce an output that cannot otherwise be achieved by single agents (22). This is for “the common good” and fosters democratic discussions where the quality of the dialogue is dependent on the quality of the information provided, together with a trusting relationship between participants (22).

Finally, optimizing and measuring how technologies are used would ensure the most effective use of technologies, and how healthcare systems could derive the greatest benefit from them. HTAs often ask medical professionals, researchers and public members of an appraisal committee to judge what patients think about a new treatment and its potential benefits and harms, ironically while restricting patient advocate and patient advocacy group input. Information directly from the source is always more reliable.

“*The regulatory and approval systems focus on efficacy of the product, not effectiveness of its use in or with people. The public is not told about this difference and often assumes that the product is effective when this has not been evaluated...”* Patient advocate (US)

## Steps to Lead Forward

Working together in partnership is transformative and can help HTA bodies to understand how to invest in active, meaningful patient engagement (22, 60). Patient participation can help to ensure that HTA agencies are aligned with the end-users [(11), https://icer.org/wp-content/uploads/2020/11/ICER_Lupus-Nephritis_Policy-Recommendations_041621.pdf].

A system to monitor and provide feed-back on how technologies are being utilized within the healthcare system, and for whom, could complete the loop for evaluating the implementation of HTA recommendations. This can create a “learning HTA and healthcare environment” that measures outcomes to inform them and builds on value over time [US Agency for Health care Research and Quality (AHRQ) https://www.ahrq.gov/learning-health-systems/index.html]. In the longer-term we would all learn to trust and benefit from availability of the most appropriate and effective technologies.

In Table 2 we have summarized our concerns together with examples of what is being done related to HTA bodies.

**Table 2:** Patient advocate and patient advocacy group concerns with examples of what is being done related to HTA bodies.

**Past concerns**	**What is happening**	**What could happen**
**Public awareness and understanding**		
Public awareness about technology development, regulation and funding including through HTAs.	COVID-19 has greatly increased public awareness about the development and regulation of medical technologies. Less so for HTA (61). CADTH Patient and Community Advisory Committee—to help explain how policies and activities impact patients, families, communities (62). https://www.cadth.ca/patient-and-community-engagement.	Continue to work on increasing public awareness—explaining processes and who is involved. Harmonize the language used. Patients can understand information when clear and visual—and sufficient data available (e.g., https://eczematherapies.com/patients/). More patient involvement and engagement at governance level. As with the HTAi Patient and Citizen Involvement Interest Group (PCIG) project: “Patient participation at the organizational level in HTA”. https://htai.org/interest-groups/pcig/projects/current-projects/.
Guidance and transparent policies on prioritization of technologies, and in developing new technologies—so that it is not largely dictated by what industry has “to offer”; or what governments “want to buy.”	National medicines policies (63). Prioritization project in South Korea (64).	Open access to information looking at global market access to health technologies for different health conditions. ICER is to publish their updated process and experience with patient advocates and patient groups (personal correspondence with their Vice President, Patient Engagement).
Diversity and health equity, account for vulnerabilities.	CADTH Patient and Citizen Advisory Committee (62).	
Need for emphasis on “value to patients,” their “unmet needs” and major concerns; attention to and consideration of care bundles and not just the technologies in isolation.	ICHOM (https://www.ichom.org/), H20 (https://health-outcomes-observatory.eu/). All CAN (https://www.all-can.org/efficiency-hub/) ICER (e.g., lupus nephritis): https://icer.org/wp-content/uploads/2020/11/ICER_Lupus-Nephritis_Policy-Recommendations_041621.pdf	Through wide use of carefully selected and developed patient-reported outcomes. In Spain, consensus expert recommendations representing all stakeholders in AMPHOS (https://sedisa.net/wp-content/uploads/2019/12/informe_de_AMPHOS-07-2.pdf) and other initiatives. In specific pathologies, measure quality of care taking into account different dimensions: CUE (65) in inflammatory bowel disease (IBD), not publicly funded.
**Patient input**		
Requests for patient input as comments or submissions, often made too late to contribute effectively to the HTA process.	KCE—input into assessment (66). UK—in scoping for an HTA (24). Guidance on how to involve patients in HTA in the Spanish Network RedETS are presented in a flowchart. Patient organizations or expert patients can participate in protocol development, outcomes' identification, assessment process, and report review (67).	Establish well-trained and selected “patient involvement reference group” at HTA management level to work collaboratively with HTA professionals and the patient and public involvement team (where it exists). Medical professionals included, particularly those experienced in shared decision making and person-centered health care. Work with researchers and HTA professionals to improve methodologies for patient/patient group input at all stages of the HTA process.
**Information for patient advocacy groups to develop patient input**		
Keep patient advocacy groups informed, e.g., if a technology is too expensive to recommend for funding; and its likely place in a care plan i.e., if there are a number of similar technologies already.	Patient groups may only be presented with “part of the story,” which can create mistrust. When invited to participate, data provided is full of acronyms and tables, with no guidance on its use. ICER Lupus nephritis summary recommendations (as above).	Working with the concept of patient and clinician driven “hope” and its place in the value assessment and use of health technologies.
Patient advocates and patient advocacy groups may find it difficult to develop the skill set and support for their work in HTAs. The training sessions that are available may be general or limited to particular aspects.	Training programs run for example by the FDA in the USA, EUPATI and WECAN in the European Union, INVOLVE in UK. The training is theoretical—still a need for manuals and other support materials (checklists, examples) to guide and assist people.	Enlist “patient coordinators” and “patient partners” (60) to provide peer support; build on skills including critical appraisal of clinical trials and other data; preparation and analysis of own data. Build “patient involvement reference group.” Publications from patient advocacy groups.
Patient advocates and patient groups may not have ready access to clinical trial and economic data for the new technology.	Lay summaries provided (58). The new drug evaluation system in Spain (REvalMed) (68) sends economic comparisons, efficacy and safety data to the patient associations consulted. This is making it easier for us to provide our feedback.	Access to full summaries of clinical trial and economic data; and how the data analyzed in an assessment, and on what basis.
**Incorporating patient input into HTAs**		
Difficulties in incorporating patient input into appraisal committee papers.	We built as a “pilot” a simple and inexpensive multi-criteria decision analysis (MCDA) framework so that patient associations could analyse and compare the value of treatments (69).	Encourage research on methodologies that would strengthen patient input and bring it into the HTA process. Support sound methodologies for patient involvement and data collection. ICER https://icer.org/work-with-icer/patients/.
We need new methods for collecting data to inform patient input into HTAs. Patient advocates and patient advocacy groups may not be funded to gather data. If they receive any funding, could create conflicts of interest.	Use of PROs and digital technologies such as apps to collect data on a disease and its treatment. Quality of life measures used as numerical tools to estimate utility and population data rather than giving a true measure of what the patient is experiencing; and without including the career.	IMI H2O open data project—important that all stakeholders have access to the same data to validate or refute the information. Projects like H20 offer this advantage. (https://health-outcomes-observatory.eu/). NICE review of methodologies (https://indepth.nice.org.uk/methods-review/index.html).
Not all patient advocates are active members of disease-specific patient advocacy groups; and not all patient support groups or charities advocate on behalf of patients as individuals. This can be a serious problem.	Some countries such as Australia accept input from individual patients, careers etc. as well as from patient groups*. The EMA does an assessment of the person by verifying their capacity and evaluating the evidence they provide to lend credibility to their discourse.	
**Follow up of funding decisions**		
Follow up of how technologies are utilized in clinical practice, if their use is directed to patients who can benefit from them, if associated with added expenses; and how the care pathway enables optimal use. We want good decisions about access to and affordability of technologies.	Valtermed** in Spain for higher-priced drugs, an access and tracking mechanism to monitor the outcomes the drugs achieve (and set pricing and payment methodology, pay-for-outcome). Registries in Italy and clinical audits (70).	A decalogue of “Quality of Care” indicators from the patient's point of view, the IQCARO project (71). Registries in Italy also used with Covid-19 (personal communication).

* *Available at: https://www1.health.gov.au/internet/hta/publishing.nsf/Content/consumers*.

** *Rosa F Valtermed: la conexión y el registro de resultados clínicos ya es posible. (2019). Available online from: https://www.diariofarma.com/2019/07/22/valtermed-la-conexion-y-el-registro-de-resultados-clinicos-ya-es-posible*.

## Conclusions

Patient advocates and patient advocacy group leaders share common interests and goals with HTA bodies regarding good decisions about access to, and affordability of health technologies. Greater benefit and effectiveness can be generated by integrating patient advocates and patient advocacy groups into HTAs, rather than treating them as separate from decision-making bodies. Good progress is being made by the HTA community. It is now time to develop consistent emerging practices globally, and to measure the results of HTA recommendations in ways that benefit the health and welfare of patients and their communities. We call on HTA leadership to work with us to build pro-active, iterative participatory methods that engage and integrate patient input into the technology development and HTA continuum. Our shared commitment can positively affect the common good as well as provide benefits to individual patients and their communities.

## Definition of Terms as Used in This Paper

*Co-creation, co-design and co-production:* Terms used interchangeably in this document to describe equal status partnerships between patient leaders and HTA bodies.

*Democratization of evidence:* Developing a better understanding and use of evidence.

*Legitimacy:* Where “democratic legitimacy” incorporates a broader view of evidence to inform efficacy, utility, and effectiveness; through inclusion and equity of allocation of resources.

“Scientific legitimacy” involves the application of scientific rigor and objectivity, leading to scientific policy goals rather than population goals.

*Healthcare technologies:* Services, diagnostics, medicines, medical devices and digital devices for use in health care.

*Patient input:* Includes patient advocates/patient group representatives on a committee, patient experts presenting at a committee meeting, and patient and patient group submissions for an HTA. This can also include caregivers.

*Patient leaders:* Patient advocates and patient advocacy groups who are active in building and strengthening patient involvement in HTAs. We describe patient leaders as people who can envision where changes to bring about solutions can take place, and work with others to enable change. McNally (72) used the term “patient leadership” to describe an investment in patient and career leaders working collaboratively in co-creation, co-design and co-production projects.

*Patient and public involvement and engagement:* A purpose of patient involvement in HTA is to improve the legitimacy of decision making; and is instrumental in producing better quality decisions that reflect patient and public preferences and values, through transparent, accountable, legitimate processes.

*Person-centered HTA:* The involvement of patients throughout the HTA process to build on patient input that has taken place in earlier stages of technology development, such as in basic research, patient preference studies, clinical trials and in being part of regulatory processes. This term was first publicly used at the plenary session “Patients at the heart of innovation” during the HTAi 2021 Annual Meeting (30).

*Service end users:* People who use the healthcare system for prevention or for treatment. Most often known as “patients.”

*Stakeholder:* Any group or individual who can affect or is affected.

## Data Availability Statement

The original contributions presented in the study are included in the article/supplementary material, further inquiries can be directed to the corresponding author.

## Author Contributions

JW, DCh, DCo, DH, RS, and ZP-W contributed to the discussions and preparation of this manuscript. All authors contributed to the article and approved the submitted version.

## Conflict of Interest

The authors declare that the research was conducted in the absence of any commercial or financial relationships that could be construed as a potential conflict of interest.

## Publisher's Note

All claims expressed in this article are solely those of the authors and do not necessarily represent those of their affiliated organizations, or those of the publisher, the editors and the reviewers. Any product that may be evaluated in this article, or claim that may be made by its manufacturer, is not guaranteed or endorsed by the publisher.

## Abbreviations

App: mobile device application
CADTH: Canadian Agency for Drugs and Technology in Health
EMA: European Medicines Agency
EUPATI: European Patients&#8217
Academy on Therapeutic Innovation
FDA: U.S. Food and Drug Administration
G-BA, The Federal Joint Committee: Germany
HTAi: health technology assessment international
ICER: Institute for Clinical and Economic Review
KCE: Belgium Health Care Knowledge Center
MHRA, Medicines and Healthcare products Regulatory Agency: UK
NICE, The National Institute for Health and Care Excellence: England
PCIG: HTAi Patient and Citizen Involvement in HTA Interest Group
PRO: patient-reported outcome
SMC: Scottish Medicines Consortium.
